# Effect of Blastocyst Morphology and Developmental Rate on Euploidy and Live Birth Rates in Preimplantation Genetic Testing for Aneuploidy Cycles With Single-Embryo Transfer

**DOI:** 10.3389/fendo.2022.858042

**Published:** 2022-04-13

**Authors:** Na Li, Yichun Guan, Bingnan Ren, Yuchao Zhang, Yulin Du, Hongjiao Kong, Yongjie Zhang, Hua Lou

**Affiliations:** Reproductive Center, The Third Affiliated Hospital of Zhengzhou University, Zhengzhou, China

**Keywords:** preimplantation genetic testing for aneuploidy, blastocyst morphology, developmental rate, euploid rates, live birth rates

## Abstract

**Objective:**

The aim of this study was to investigate whether blastocyst morphology and developmental rate are associated with euploidy and live birth rates (LBRs) in single euploid frozen–thawed embryo transfer (FET) cycles.

**Design:**

Retrospective cohort study.

**Methods:**

This study included 431 preimplantation genetic testing for aneuploidy (PGT-A) cycles followed by 393 FET cycles performed at our center from June 2017 to March 2021. All cycles were analyzed for euploidy based on blastocyst morphology (good, average and poor), developmental stage (day 5 and 6) and maternal age (< 35 and ≥ 35 years old). Multivariate logistic analysis models were used to identify the independent effects of conventional blastocyst morphology, developmental rate and morphological parameters (degree of blastocoele expansion, and grade of inner cell mass and trophectoderm (TE)) on LBRs.

**Results:**

In the group of women aged < 35 years, compared with poor-quality blastocysts, good-quality blastocysts (62.90% vs. 32.46%; odds ratio (OR) 3.163, 95% confidence interval (CI) 2.247–4.451; *P* < 0.001) and average-quality blastocysts (46.70% vs. 32.46%; OR 1.665, 95% CI 1.287–2.154; *P* < 0.001) had significantly higher euploidy rates. Additionally, day 5 blastocysts were associated with higher euploidy rates than day 6 blastocysts (49.28% vs. 35.02%; OR 1.506, 95% CI 1.191–1.903; *P*= 0.001). In the group of women aged ≥ 35 years, euploidy rates were also associated with blastocyst morphology, with 41.86%, 45.65% and 24.39% of good, average and poor-quality embryos, respectively, exhibiting euploidy. However, no relationship was seen between euploidy and blastocyst developmental rate. Multiple logistic regression analysis show that overall blastocyst morphology of euploid embryos was not associated with LBR, only embryos with A-grade TE had significantly higher LBRs than those with C-grade TE (62.71% vs. 45.40%; OR 2.189, 95% CI 1.166–4.109; *P*=0.015). Similarly, LBRs were significantly higher when day 5 blastocysts were transferred than when day 6 blastocysts were transferred (57.75% vs. 41.67%; OR 2.132, 95% CI 1.370–3.318; *P* = 0.001).

**Conclusion:**

Poor-quality embryos have reduced rates of euploidy. However, blastocyst developmental rate only significantly associates with euploidy rates in women aged younger than 35. Furthermore, only TE grade and blastocyst developmental rate are significantly associated with LBRs following FET cycles.

## Introduction

Although the development of assisted reproductive technologies (ARTs) has helped many couples to conceive, early embryo arrest and recurrent pregnancy loss remain significant challenges in the treatment of infertility (1). Recent studies have shown that more than half of human preimplantation embryos produced using *in vitro* fertilization (IVF) possess some degree of chromosomal abnormality (2). Furthermore, the proportion of aneuploid embryos increases significantly with advancing maternal age (3). Therefore, a major goal of reproductive medicine is to identify the best embryos, thereby maximizing the rate of success of IVF-derived embryo transfer.

The traditional system of embryo grading is based on the evaluation of morphological parameters, including the degree of blastocoel expansion, and the grades of inner cell mass (ICM) and trophectoderm (TE), and is widely used to select embryos with optimal development potential. However, this system cannot accurately evaluate the ploidy status of embryos, as more than half of embryos of good morphological grade are later determined as being aneuploid (4). With advances in molecular genetic techniques and improved embryo culture methods, preimplantation genetic testing for aneuploidy (PGT-A) with next-generation sequencing (NGS) may be a reliable method to improve embryo selection by identifying euploid embryos with normal chromosomes. However, controversy remains regarding the clinical effectiveness of PGT-A (5–7). A randomized controlled study suggested that PGT-A reduced the early pregnancy loss rate after the first embryo transfer (6), while a recent trial showed that PGT-A did not improve the frequency of cumulative live-birth (7). Preimplantation Genetic Diagnosis (PGD) European Consortium recommended PGT-A for advanced maternal age (AMA), recurrent implantation failure, recurrent pregnancy loss (RPL) and severe male factor (SMF) (8).

However, it remains unclear whether conventional parameters of embryo assessment, such as blastocyst morphology and developmental rate, correlate with the ploidy status of embryos. In an observational study, blastocyst morphology was found to be predictive of chromosomal status (9). Similarly, another study reported that blastocyst morphology was weakly associated with ploidy status (10). Additionally, Majumdar et al. (11) concluded that day 5 blastocysts had higher euploidy rates than day 6 blastocysts (70% vs. 34.1%). However, the relationship between blastocyst morphology, developmental rate, morphological parameters and pregnancy outcomes after the transfer of euploid blastocysts is not well understood. In 2017, Irani et al. reported that blastocyst morphologic grading, particularly ICM grade, is a valid predictor of pregnancy rate subsequent to frozen embryo transfer (FET) (12). Similarly, in another study, the live birth rates (LBRs) of day 5 euploid blastocysts were significantly higher than those of day 6 blastocysts (13). In contrast, Anderson et al. suggested that clinical outcomes were not significantly influenced by embryo morphology, provided a single euploid embryo is transferred (14).

In view of these contradictory findings, the aim of the present study was to investigate whether blastocyst morphology and developmental rate correlate with embryo euploidy or live birth rates (LBRs) following an FET cycle and the transfer of euploid embryos. Such knowledge would improve our understanding of euploid embryo selection, and assist physicians in clinical practice and patients undergoing PGT-A for conception.

## Materials and Methods

### Study Design and Population

This was a retrospective cohort study conducted at the Reproductive Center of the Third Affiliated Hospital of Zhengzhou University from June 2017 to March 2021. We only included patients who underwent first PGT-A cycles and subsequently received first single euploid FET cycles. We excluded cycles if the embryos underwent preimplantation genetic diagnosis, were donated or involved women with uterine malformation. This study was performed in accordance with the Code of Ethics in the Declaration of Helsinki and was approved by the Ethics Review Committee of our hospital (protocol number 2021-WZ-010).

### Ovarian Stimulation Protocol

Ovarian stimulation was performed using a gonadotropin-releasing hormone (GnRH) antagonist protocol in patients undergoing PGT-A cycles. Briefly, recombinant follicle-stimulating hormone (FSH; Gonal-F, Merck Serono, Switzerland) was administered on day 2 or 3 of the menstrual period, and the ovarian response was monitored by transvaginal ultrasound and determination of serum estradiol concentrations. When the diameter of the dominant follicle reached 12–14 mm, 0.25 mg of a GnRH antagonist (Cetrotide, Merck Serono, Switzerland) was injected to cause pituitary suppression. Ovulation was triggered by administration of 0.2 mg Dophereline (Ipsen Pharma Biotech, France) or 250 µg recombinant human chorionic gonadotropin (hCG; Ovidrel, Merck Serono, Switzerland). Ultrasound-guided oocyte retrieval was performed 33–36 h after the triggering of ovulation.

### Laboratory Protocols

Intracytoplasmic sperm injection (ICSI) was used for all cycles included in this study, and was carried out 4–6 h after oocyte retrieval. The presence of two equally sized pronuclei was assessed 16–18 h after insemination. All embryos were cultured to the blastocyst stage in Vitrolife sequential medium (Goteborg, Sweden). Blastocyst evaluation was performed prior to embryo biopsy. Embryologists graded the blastocysts based on the degree of expansion and the morphology of ICM and TE (15). The degree of expansion included the following six grades:(1) a nonexpanded embryo with the blastocele filling <50%; (2) the blastocele filling >50% of the embryo; (3) a full blastocyst with a blastocoele filling the embryo; (4) an expanded blastocyst with a blastocoele volume larger than that of the full blastocyst, with a thinning zona; (5) a hatching blastocyst with the TE starting to herniate through the zona; (6) a hatched blastocyst, with the blastocyst completely escaping from the zona. The ICM was graded as follows: (A) tightly packed with many cells, (B) loosely gathered with several cells or (C) very few cells. The TE was assigned one of the following grades: (A) many cells forming a cohesive epithelium, (B) few cells establishing a loose epithelium or (C) very few large cells. In our center, only blastocysts with ICM grade of at least B were biopsied. We divided blastocysts into three quality groups based mainly on ICM and TE grades: good (4AA, 5AA, 6AA, 4AB, 5AB, 6AB, 4BA, 5BA and 6BA), average (4BB, 5BB and 6BB) and poor (4AC, 5AC, 6AC, 4BC, 5BC and 6BC). Next, the embryos were biopsied on day 5 or 6, depending on the time of blastulation. Day 5 blastocysts were defined as “faster growing” and day 6 blastocysts as “slower growing.” All embryo grading was carried out by two experienced embryologists to ensure consistency. TE biopsies were performed as previously described (16). If patients obtained more than 6 blastocysts, only 6 blastocysts are generally biopsied for economic reasons. NGS on the NextSeq550 platform (Illumina Inc., San Diego, CA), a widely used technology for 24-chromosome aneuploidy screening based on quantitative polymerase chain reaction (17), was used to diagnose embryos as euploid, aneuploid or mosaic. Finally, the biopsied blastocysts were cryopreserved using the Kitazato-based vitrification method (18).

### Endometrial Preparation

Endometrial preparation FET included natural cycles or hormone replacement therapy (HRT) cycles. In general, women with regular menstrual cycles underwent natural FET cycles in which transvaginal ultrasound was used from day 10 of the menstrual cycle to monitor the development of the dominant follicle and endometrial thickness. Embryo transfer was performed on day 5 after a positive urinary urine luteinizing hormone test. Subsequently, 10 mg oral dydrogesterone (Duphaston; Solvay Pharmaceuticals BV) was administered three times daily from the day after ovulation to support the luteal phase. Patients who underwent HRT FET cycles took 4–8 mg estradiol valerate (ValieraVR; Laboratories Recalcine) daily for 12 days from day 3 of the menstrual cycle. When the endometrium measured ≥ 7 mm in thickness, as monitored by transvaginal ultrasound, twice-daily 10 mg oral dydrogesterone (Duphaston; Solvay Pharmaceuticals BV) and daily 90 mg progesterone sustained-release vaginal gel (Xenotong, Merck Sherano, Switzerland) daily was initiated. On day 6 of progesterone administration, a single euploid embryo was transferred. If pregnancy occurred, luteal support was continued until 7 weeks of gestation.

### Outcome Measures

Primary outcomes were euploidy rates and LBRs. Women receiving PGT-A cycles were divided into two age groups: < 35 and ≥ 35 years old. Euploidy rates were compared for different blastocyst morphology (good, average and poor) and developmental rates (days 5 and 6) within the same age group. Euploidy rates were calculated as the number of euploid embryos with 46 chromosomes divided by the total number of biopsied embryos with genetic results. The primary endpoint was LBRs after single-euploid FET. The LBRs were defined as the number of live births divided by the sum of embryo transfer cycles included in the cohort.

### Statistical Analysis

All statistical analyses were performed using SPSS 25.0 statistical software (IBM, United States) and the figures were produced using GraphPad Prism 9 software. Continuous variables were tested for normality and are expressed as means ± standard deviations, and were compared using Student’s *t*-tests or Mann–Whitney *U* tests. Categorical variables are shown as percentage frequencies, and chi-squared tests were performed to identify statistically significant differences. The association between blastocyst morphology and developmental rate and the occurrence of euploidy was studied using generalized estimating equations analysis to adjust for the embryos derived from the same patient. Multivariate logistic analysis was used to investigate the effect of blastocyst morphology, developmental rate and morphological parameters on LBRs. Odds ratios (ORs) with 95% confidence intervals (CIs) were calculated to control for confounding factors. *P* < 0.05 was considered statistically significant.

## Results

Four hundred and thirty-one PGT-A cycles, during which 1,872 available blastocysts were generated and 1,647 embryos were biopsied for ploidy status and which were followed by 393 first FET cycles, were included in the data analysis. The mean maternal age of the patients was 32.04 ± 5.18 years. The embryological characteristics and clinical outcomes are listed in **Table 1**. Forty-seven of the biopsied blastocysts gave no genetic result (2.85%) due to failed amplification. The overall euploidy, aneuploidy and mosaic rates of the embryos were 40.44%, 47.13% and 12.44%, respectively.

**Table 1:** Embryological and clinical outcomes in patients who underwent PGT-A cycles.

Characteristic	Description
Number of PGT-A cycles (n)	431
Maternal age (years)	32.04 ± 5.18
Maternal BMI (kg/m^2^)	23.81 ± 3.05
Duration of infertility (years)	2.90 ± 1.99
Type of infertility, n (%)	
Primary	108 (25.06)
Secondary	323 (74.94)
Infertility diagnosis, n (%)	
Tubal factor	155 (35.96)
Male factor	126 (29.23)
Diminished ovarian reserve	61 (14.15)
Combined factor	64 (25.06)
Unexplained factor	25 (5.80)
Number of prior pregnancies (n)	2.07 ± 1.82
Number of prior embryos transfer (n)	1.28 ± 0.65
Number of prior successful transfer (n)	0.67 ± 0.50
Indication for PGT-A, n (%)	
AMA	45 (10.44)
RIF	89 (20.65)
RPL	111 (25.75)
Combined indication	186 (43.16)
Basal FSH	6.57 ± 2.16
Number of oocytes retrieved (n)	6,481
Number of mature oocytes (n)	4,885
Number of 2PN cleavages (n)	3,997
Number of available blastocysts generated	1872
Number of blastocysts biopsied (n)	1,647
Blastocysts with no genetic results (n,%)	47 (2.85)
Results of 1,600 blastocysts biopsied (n, %)	
Euploid	647 (40.44)
Aneuploid	754 (47.13)
Mosaic	199 (12.44)
Number of euploid FETs (n,%)	393
Implantation rate	234/393 (59.54)
Miscarriage rate	36/234 (15.38)
Live birth rate	198/393 (50.38)

Values are presented as means ± standard deviations or n (%). BMI, body mass index; PGT-A, preimplantation genetic testing for aneuploidy; FSH, follicle-stimulating hormone; AMA, advanced maternal age; RIF, recurrent implantation failure; RPL, recurrent pregnancy loss.

Euploidy rates for all age groups are shown in **Supplementary Figure**. The generalized estimating equations analysis revealed that compared with poor-quality blastocysts, good-quality blastocysts (60.00% vs. 31.64%; OR 2.900, 95% CI 2.130–3.948; *P* < 0.001) and average-quality blastocysts (47.98% vs. 31.64%; OR 1.828, 95% CI 1.445–2.312; *P* < 0.001) had significantly higher euploidy rates after adjusting for blastocyst developmental rate, maternal age, maternal BMI, duration of infertility, type of infertility, infertility diagnosis, number of prior pregnancies, indication for PGT-A and basal FSH. Besides, day 5 blastocysts were associated with higher euploidy rates than day 6 blastocysts (48.49% vs. 34.72%; OR 1.431, 95% CI 1.155–1.772; *P*= 0.001) after adjusting for blastocyst morphology, maternal age, maternal BMI, duration of infertility, type of infertility, infertility diagnosis, number of prior pregnancies, indication for PGT-A and basal FSH. Meanwhile, in order to eliminate the embryos derived from the same patient, we performed generalized estimating equations analysis to further assess the association between blastocyst morphology/developmental rate and euploidy rate. After adjusted for blastocyst morphology, developmental rate, maternal age, maternal BMI, duration of infertility, type of infertility, infertility diagnosis, number of prior pregnancies, indication for PGT-A and basal FSH, blastocyst morphology and developmental rate are still associated with euploidy rate (**Supplemental Table**).

**Figure 1:**
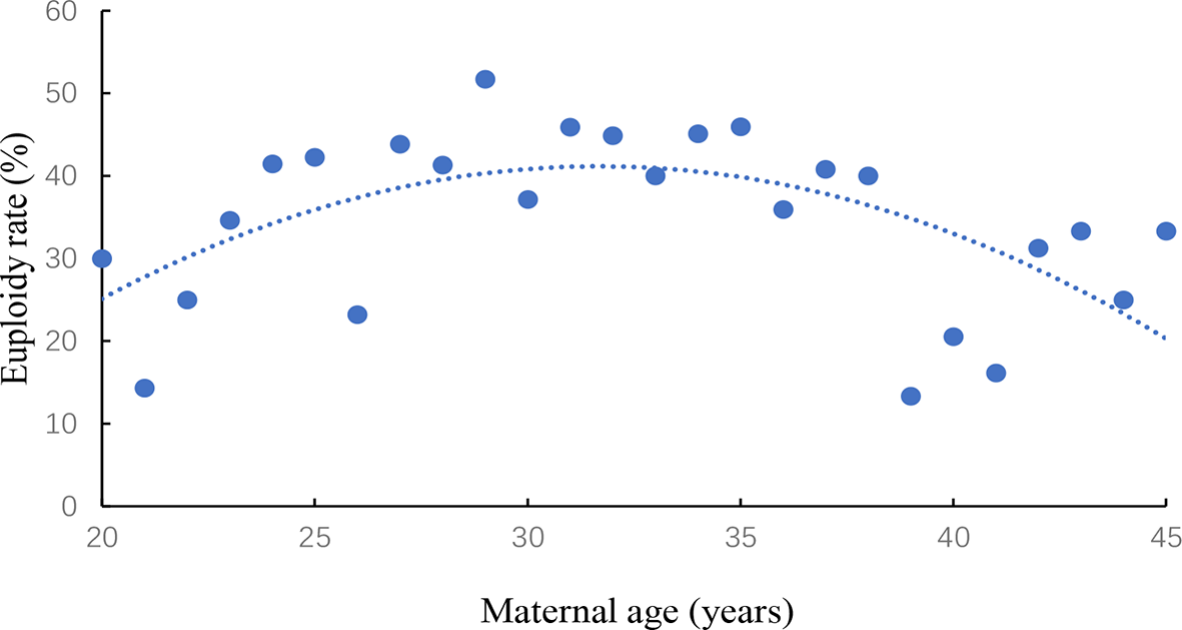
Embryo euploidy according to women’s age.

The association between women’s age and euploidy rate was evaluated in **Figure 1**. Euploidy rate was start to gradually decline in women older than 35 years of age.

**Figure 2:**
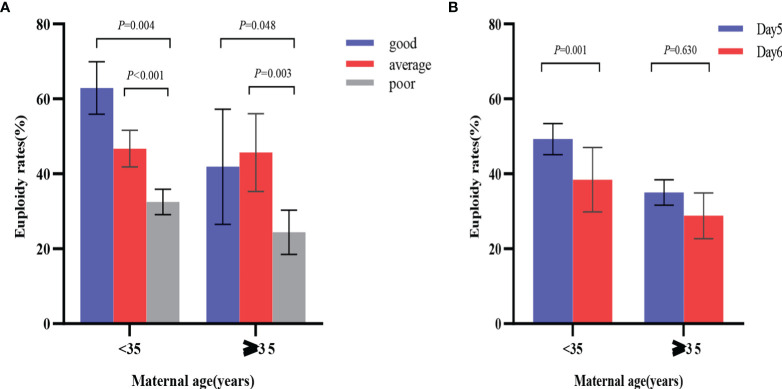
Comparison of euploidy rates by blastocyst morphology and developmental rates in the two age groups. **(A)** odds ratio was adjusted for blastocyst developmental rate, maternal age, maternal BMI, duration of infertility, type of infertility, infertility diagnosis, number of prior pregnancies, indication for PGT-A and basal FSH; **(B)** odds ratio was adjusted for blastocyst morphology, maternal age, maternal BMI, duration of infertility, type of infertility, infertility diagnosis, number of prior pregnancies, indication for PGT-A and basal FSH. *P* values are adjusted *p* values.

Euploidy rates for the two different age groups are shown in **Figure 2**. In the < 35 years age group, blastocyst morphology was not associated with the age of patients, and the corresponding age of good, average and poor-quality embryos were 29.40±2.86, 29.27±2.83 and 29.09±3.01, respectively (*P*=0.349). Compared with poor-quality blastocysts (236/727), good-quality blastocysts (117/186) (62.90% vs. 32.46%; OR 2.818, 95% CI 1.401–5.665; *P*=0.004) and average-quality blastocysts (184/394) (46.70% vs. 32.46%; OR 1.669, 95% CI 1.286–2.166; *P*< 0.001) yielded significantly higher euploidy rates after adjusting for blastocyst developmental rate, maternal age, maternal BMI, duration of infertility, type of infertility, infertility diagnosis, number of prior pregnancies, indication for PGT-A and basal FSH. Additionally, day 5 blastocysts (274/556) were associated with higher rates of euploidy than day 6 blastocysts (263/751) (49.28% vs. 35.02%; OR 1. 496, 95% CI 1.181–1.894; *P* = 0.001) after adjusting for blastocyst morphology, maternal age, maternal BMI, duration of infertility, type of infertility, infertility diagnosis, number of prior pregnancies, indication for PGT-A and basal FSH.

Similarly, in the ≥ 35 years age group, compared with poor-quality blastocysts (50/205), good-quality blastocysts (18/43) (41.86% vs. 24.39%; OR 2.131, 95% CI 1.071–4.242; *P* = 0.048) and average-quality blastocysts (42/92) (45.65% vs. 24.39%; OR 2.722, 95% CI 1.566–4.732; *P* = 0.003) had significantly higher euploidy rates after adjusting for developmental rate, maternal age, maternal BMI, duration of infertility, type of infertility, infertility diagnosis, number of prior pregnancies, indication for PGT-A and basal FSH. However, the difference in euploidy rates between the day 5 (48/125) and day 6 (62/215) blastocysts was not statistically significant (38.40% vs. 28.84%; OR = 1.135, 95% CI 0.677–1.903; *P* = 0.630) after adjusting for blastocyst morphology, maternal age, maternal BMI, duration of infertility, type of infertility, infertility diagnosis, number of prior pregnancies, indication for PGT-A and basal FSH.

Only 393 cycles progressed to first single-euploid FET; 198 of these ended with live birth and 195 did not achieve live birth. The characteristics of patients who underwent FET cycles are summarized in **Table 2**. The possibility of live birth was not significantly influenced by maternal age, duration of infertility, type of infertility, infertility diagnosis, number of prior pregnancies, number of prior embryos transfer, number of prior successful transfer, indications for PGT-A or basal FSH (*P* > 0.05). The proportions of natural FET cycles and maternal body mass index (BMI) were significantly higher in the no live birth group than in the live birth group (*P* < 0.05). Endometrial thickness on transfer day was also found to be significantly lower in the no live birth group than in the live birth group (*P* < 0.05).

**Table 2:** Patient characteristics of transferred frozen-thawed euploid blastocysts in terms of birth outcome.

Parameters	Live birth (*n* = 198)	No live birth (*n* = 195)	*P* value
Maternal age (years)	31.12 ± 4.76	32.05 ± 4.70	0.052
Maternal BMI (kg/m^2^)	23.77 ± 3.06	24.64 ± 3.00	0.005
Duration of infertility (years)	2.78 ± 1.89	2.94 ± 2.09	0.425
Type of infertility, n (%)			0.245
Primary	58 (29.29)	47 (24.10)	
Secondary	140 (70.71)	148 (75.90)	
Infertility diagnosis, n (%)			0.979
Tubal factor	74 (37.37)	70 (35.90)	
Male factor	57 (28.79)	60 (30.77)	
Diminished ovarian reserve	27 (13.64)	29 (14.87)	
Combined factor	29 (14.65)	26 (13.33)	
Unexplained factor	11 (5.56)	10 (5.13)	
Number of prior pregnancies (n)	1.87 ± 1.68	2.22 ± 1.97	0.065
Number of prior embryos transfers (n)	1.25 ± 0.63	1.23 ± 0.51	0.706
Number of prior successful transfers (n)	0.60 ± 0.55	0.56 ± 0.52	0.908
Indication for PGT-A, n (%)			0.411
AMA	15 (7.58)	23 (11.79)	
RIF	44 (22.22)	36 (18.46)	
RPL	52 (26.26)	46 (23.59)	
Combined indications	87 (43.94)	90 (47.18)	
Basal FSH (IU/L)	6.38 ± 2.34	6.28 ± 2.47	0.683
Endometrial preparation protocols, n (%)			0.022
Natural	94 (47.47)	115 (58.97)	
HRT	104 (52.53)	80 (41.03)	
Endometrial thickness on transfer day (mm)	9.32 ± 1.53	8.82 ± 1.54	0.001

Values are presented as means ± standard deviations or n (%). BMI, body mass index; PGT-A, preimplantation genetic testing for aneuploidy; FSH, follicle-stimulating hormone; AMA, advanced maternal age; RIF, recurrent implantation failure; RPL, recurrent pregnancy loss; HRT; hormone replacement therapy.

Finally, to determine whether conventional blastocyst morphology, developmental rate and morphological parameters influenced LBRs, a multivariate logistic regression was performed after adjusting for confounding factors (maternal age, maternal BMI, type of infertility, infertility diagnosis, number of prior pregnancies, endometrial preparation protocols and endometrial thickness on transfer day), as shown in **Table 3**. We found that the odds of a live birth were significantly higher for A-grade TE blastocysts than for C-grade TE blastocysts (62.71% vs. 45.40%; OR 2.212, 95% CI 1.164–4.201; *P* = 0.015). Moreover, the likelihood of live birth was significantly higher for day 5 euploid blastocysts compared to day 6 euploid blastocysts (57.75% vs. 41.67%; OR 2.247, 95% CI 1.460–3.460; *P*<0.001). However, blastocyst morphology, the degree of blastocoele expansion and ICM grade were not significantly associated with LBRs.

**Table 3:** Multiple logistic regression analysis of the association between live birth rate, blastocyst grade and developmental rates in single-euploid embryo transfer cycles.

Variable	Value	Live birth (%)	OR (95% CI)	*P* value
Blastocyst				
	Good	55.56 (50/90)	1.689 (0.980–2.909)	0.059
	Average	53.49 (69/129)	1.423 (0.886–2.287)	0.145
	Poor	45.40 (79/174)		
ICM				
	A	56.06 (37/66)	1.540 (0.875–2.711)	0.134
	B	49.24 (161/327)		
TE				
	A	62.71 (37/59)	2.212 (1.164–4.201)	0.015
	B	51.25 (82/160)	1.341 (0.857–2.099)	0.199
	C	45.40 (79/174)		
Developmental rate				
	Day 5	57.75 (123/213)	2.247 (1.460–3.460)	<0.001
	Day 6	41.67 (75/180)		

OR, odds ratio; CI, conﬁdence interval; ICM, inner cell mass; TE, trophectoderm. The values for LBRs are adjusted for maternal age; maternal body-mass index; type of infertility; infertility diagnosis; number of prior pregnancies; endometrial preparation protocols and endometrial thickness on embryo transfer day.

## Discussion

This study determined the effects of blastocyst morphology and developmental rate on embryo euploidy and LBRs following FET cycles. We found that compared to poor-quality embryos, both good and average-quality embryos yield significantly higher euploidy rates for patients of the same age group. We also found that faster-growing embryos (day 5) have a greater likelihood of euploidy than slower-growing embryos (day 6), especially when maternal age is less than 35 years. TE grade and developmental rate had the strongest association with live birth.

It is well known that maternal age is a significant factor affecting pregnancy outcomes, both in ART and spontaneous conceptions. The age-related decline in reproductive ability is attributed to a decline in ovarian reserve and an increase in aneuploidy rates (19). Given this, we stratified the patients by age group to investigate whether blastocyst morphology and developmental rate are associated with euploidy rates. We found that poor-quality embryos had a reduced rate of euploidy compared to good and average-quality embryos from within the same age group. This finding is in agreement with a recent study that reported embryos with better morphological scores showed a higher euploidy rate than embryos of lower quality (9). Nevertheless, traditional morphology based selection should not be relied on to ensure transfer of euploid blastocysts, as a significant proportion of aneuploid embryos exhibit good morphology according to the criteria used.

Another embryological parameter analyzed in this study was the developmental rate at which each embryo reached the blastocyst stage. Embryos reaching the expanded blastocyst stage by day 5 had higher euploidy rates than those that did so by day 6. However, the timing of blastocyst formation was not linked to chromosomal ploidy status in women aged older than 35. We therefore suggest that morphology is a more reliable parameter than the speed of blastocyst formation for predicting embryo chromosomal status, especially in AMA women.

We found that the possibility of live birth was not significantly influenced by maternal age. Consistent with Irani et al. previously published study, maternal age did not influence the implantation potential of euploid embryos (20). However, Reig et al. demonstrated that increasing maternal age is associated with diminished live birth rates when analyzing only euploid embryo transfers (21). The effects of blastocyst morphology and developmental rate on LBRs can be interpreted as an indirect indicator of their effects on embryo development and implantation potential. It seems reasonable to assume that once euploid blastocysts are transferred, pregnancy outcome is independent of blastocyst morphology. A recent retrospective cohort study showed that maternal age, blastocyst morphology and day of biopsy are all associated with sustained implantation rate even after euploid embryos are transferred (22). Indeed, we found that blastocyst morphology is not associated with embryo survival: the LBRs of poor-quality euploid blastocysts were comparable to those of blastocysts scored as good or average quality. Thus, morphology does not appear to be reliable predictor of live birth if multiple euploid embryos are available for transfer.

The LBRs of embryos with different developmental rates were found to be significantly different, suggesting that embryo developmental rate correlates with survival potential. Our findings are consistent with the results of Irani et al., who reported that day 5 euploid embryos had higher LBRs than day 6 embryos (13). This may reflect a higher survival rate of faster developing (i.e., day 5) embryos or improved synchronization between the embryo and the endometrium on day 5. However, in contrast, a retrospective cohort study demonstrated that day 5 and 6 euploid embryos yielded the same LBRs (23).

It is widely known that the Gardner and Schoolcraft grading system consists of three criteria: the degree of blastocoel expansion and the qualities of the ICM and TE (24). Previous research findings conflict on which parameters are associated with blastocyst transfer outcomes. Some studies have suggested that ICM quality might be a key factor in determining the LBR (25, 26). Indeed, as the ICM itself contributes to the fetal tissue, it is theorized that ICM grade should be the most important morphological feature influencing transfer outcomes. However, a recent publication reported that blastocoele expansion is the most significant morphological predictor of live birth after single blastocyst FET (27). In the current retrospective cohort study, we found that LBRs for A-grade TE euploid blastocysts are higher than those for C-grade TE euploid blastocysts, suggesting that TE grade influences embryo developmental competence. In line with this, numerous studies have reported that TE grade can be used as an independent and accurate predictor of pregnancy outcomes (28–30). The TE differentiates into the placenta, which requires healthy TE to be capable of invading the endometrium and initiating the complex implantation process required to maintain a healthy pregnancy. Additionally, it was shown that embryos with higher TE grades secrete hCG earlier than those with lower TE grades, with such secretion being essential for embryo implantation and embryo–endometrium cross-talk (31).

As all PGT-A and FET cycles included in this study were performed at a single reproductive center, the reliability of our results is increased. Second, embryo scoring was performed by two experienced embryologists, each of whom had 5 years of experience. Third, we only included single-euploid embryo transfer cycles, which may have eliminated additional confounding factors that might influence outcomes. However, this study also has some limitations. First, a retrospective cohort study design has its inherent limitations. Second, if more than one euploid embryo is available for transfer, blastocysts with good quality are prioritized, which might have led to selection bias. Additional prospective studies of larger sample size are required to validate our current findings.

## Conclusion

In conclusion, euploidy rates after NGS were found to be lower for poor-quality embryos than for average-quality or good-quality embryos. Increased rates of euploidy were seen for faster-developing (i.e. day 5) embryos but only in women aged younger than 35. Moreover, blastocyst developmental rate and TE grade were associated with the highest probability of live birth after transfer of a single euploid embryo.

## Data Availability Statement

The raw data supporting the conclusions of this article will be made available by the authors, without undue reservation.

## Ethics Statement

The studies involving human participants were reviewed and approved by the Third Affiliated Hospital of Zhengzhou University. Written informed consent for participation was not required for this study in accordance with the national legislation and the institutional requirements.

## Author Contributions

HL and YG proposed the study. NL, BR, and YCZ acquired and analyzed the data. YD, HK, and YJZ prepared all tables and figures. NL wrote the manuscript. HL revised the manuscript. All authors read and approved the final manuscript.

## Funding

This study was supported by grant 2018020198 from Henan Medical Science and Technology Research Project, China.

## Conflict of Interest

The authors declare that the research was conducted in the absence of any commercial or financial relationships that could be construed as a potential conflict of interest.

## Publisher’s Note

All claims expressed in this article are solely those of the authors and do not necessarily represent those of their affiliated organizations, or those of the publisher, the editors and the reviewers. Any product that may be evaluated in this article, or claim that may be made by its manufacturer, is not guaranteed or endorsed by the publisher.

## Abbreviations

LBRs, live birth rates; FET, frozen–thawed embryo transfer; NGS, next-generation sequencing; PGT-A, preimplantation genetic testing for aneuploidy; ICSI, intracytoplasmic sperm injection; ICM, inner cell mass; TE, trophectoderm; AMA, advanced maternal age; RIF, recurrent implantation failure, RSA, recurrent spontaneous abortion; BMI, body mass index; FSH, follicle stimulating hormone; LH, luteinizing hormone; HRT, hormone replacement therapy; 2PN, two pronuclear.

## References

[1] Lemos E Zhang D Van Voorhis B Hu X. Healthcare Expenses Associated With Multiple vs Singleton Pregnancies in the United States. Am J Obstet Gynecol (2013) 209(6):586.e1–.e11. doi: 10.1016/j.ajog.2013.10.005 24238479

[2] Macklon N Geraedts J Fauser B. Conception to Ongoing Pregnancy: The 'Black Box' of Early Pregnancy Loss. Hum Reprod Update (2002) 8(4):333–43. doi: 10.1093/humupd/8.4.333 12206468

[3] Steiner AZ Jukic AM. Impact of Female Age and Nulligravidity on Fecundity in an Older Reproductive Age Cohort. Fertil Steril (2016) 105(6):1584–8 e1. doi: 10.1016/j.fertnstert.2016.02.028 26953733 PMC4893975

[4] Minasi M Colasante A Riccio T Ruberti A Casciani V Scarselli F. Correlation Between Aneuploidy, Standard Morphology Evaluation and Morphokinetic Development in 1730 Biopsied Blastocysts: A Consecutive Case Series Study. Hum Reprod (Oxf Engl) (2016) 31(10):2245–54. doi: 10.1093/humrep/dew183 27591227

[5] Munne S Kaplan B Frattarelli JL Child T Nakhuda G Shamma FN. Preimplantation Genetic Testing for Aneuploidy Versus Morphology as Selection Criteria for Single Frozen-Thawed Embryo Transfer in Good-Prognosis Patients: A Multicenter Randomized Clinical Trial. Fertil Steril (2019) 112(6):1071–79. doi: 10.1016/j.fertnstert.2019.07.1346 31551155

[6] Rubio C Bellver J Rodrigo L Castillón G Guillén A Vidal C. *In Vitro* Fertilization With Preimplantation Genetic Diagnosis for Aneuploidies in Advanced Maternal Age: A Randomized, Controlled Study. Fertil Steril (2017) 107(5):1122–9. doi: 10.1016/j.fertnstert.2017.03.011 28433371

[7] Yan J Qin Y Zhao H Sun Y Gong F Li R. Live Birth With or Without Preimplantation Genetic Testing for Aneuploidy. N Engl J Med (2021) 385(22):2047–58. doi: 10.1056/NEJMoa2103613 34818479

[8] Harper J Wilton L Traeger-Synodinos J Goossens V Moutou C SenGupta S. The ESHRE PGD Consortium: 10 Years of Data Collection. Hum Reprod Update (2012) 18(3):234–47. doi: 10.1093/humupd/dmr052 22343781

[9] Capalbo A Rienzi L Cimadomo D Maggiulli R Elliott T Wright G. Correlation Between Standard Blastocyst Morphology, Euploidy and Implantation: An Observational Study in Two Centers Involving 956 Screened Blastocysts. Hum Reprod (Oxf Engl) (2014) 29(6):1173–81. doi: 10.1093/humrep/deu033 24578475

[10] Alfarawati S Fragouli E Colls P Stevens J Gutiérrez-Mateo C Schoolcraft W. The Relationship Between Blastocyst Morphology, Chromosomal Abnormality, and Embryo Gender. Fertil Steril (2011) 95(2):520–4. doi: 10.1016/j.fertnstert.2010.04.003 20537630

[11] Majumdar G Majumdar A Verma I Upadhyaya K. Relationship Between Morphology, Euploidy and Implantation Potential of Cleavage and Blastocyst Stage Embryos. J Hum Reprod Sci (2017) 10(1):49–57. doi: 10.4103/0974-1208.204013 28479756 PMC5405648

[12] Irani M Reichman D Robles A Melnick A Davis O Zaninovic N. Morphologic Grading of Euploid Blastocysts Influences Implantation and Ongoing Pregnancy Rates. Fertil Steril (2017) 107(3):664–70. doi: 10.1016/j.fertnstert.2016.11.012 28069172

[13] Irani M O'Neill C Palermo G Xu K Zhang C Qin X. Blastocyst Development Rate Influences Implantation and Live Birth Rates of Similarly Graded Euploid Blastocysts. Fertil Steril (2018) 110(1):95–102.e1. doi: 10.1016/j.fertnstert.2018.03.032 29908774

[14] Anderson R Whitney J Schiewe M. Clinical Benefits of Preimplantation Genetic Testing for Aneuploidy (PGT-A) for All *In Vitro* Fertilization Treatment Cycles. Eur J Med Genet (2020) 63(2):1–8. doi: 10.1016/j.ejmg.2019.103731 31362121

[15] Gardner DK Surrey E Minjarez D Leitz A Stevens J Schoolcraft WB. Single Blastocyst Transfer: A Prospective Randomized Trial. Fertil Steril (2004) 81(3):551–5. doi: 10.1016/j.fertnstert.2003.07.023 15037401

[16] Lou H Li N Guan Y Zhang Y Hao D Cui S. Association Between Morphologic Grading and Implantation Rate of Euploid Blastocyst. J Ovarian Res (2021) 14(1):18–26. doi: 10.1186/s13048-021-00770-8 33485390 PMC7827997

[17] Zimmerman R Tao X Marin D Werner M Hong K Lonczak A. Preclinical Validation of a Targeted Next Generation Sequencing-Based Comprehensive Chromosome Screening Methodology in Human Blastocysts. Mol Hum Reprod (2018) 24(1):37–45. doi: 10.1093/molehr/gax060 29186554

[18] Kato K Ueno S Yabuuchi A Uchiyama K Okuno T Kobayashi T. Women’s Age and Embryo Developmental Speed Accurately Predict Clinical Pregnancy After Single Vitrified-Warmed Blastocyst Transfer. Reprod BioMed Online (2014) 29:411–6. doi: 10.1016/j.rbmo.2014.06.007 25129691

[19] Sunderam S KD Crawford SB Folger SG Jamieson DJ Warner L. Assisted Reproductive Technology Surveillance - United States, 2012. MMWR Surveill Summ (2015) 64:1–29. doi: 10.15585/mmwr.ss6411a1 26270152

[20] Irani M Zaninovic N Rosenwaks Z Xu K. Does Maternal Age at Retrieval Influence the Implantation Potential of Euploid Blastocysts? Am J Obstet Gynecol (2019) 220(4):379.e1–.e7. doi: 10.1016/j.ajog.2018.11.1103 30521800

[21] Reig A Franasiak J Scott RT Jr. Seli E. The Impact of Age Beyond Ploidy: Outcome Data From 8175 Euploid Single Embryo Transfers. J Assist Reprod Genet (2020) 37(3):595–602. doi: 10.1007/s10815-020-01739-0 32173784 PMC7125286

[22] Awadalla MS Vestal NL McGinnis LK Ahmady A Paulson RJ. Effect of Age and Morphology on Sustained Implantation Rate After Euploid Blastocyst Transfer. Reprod BioMed Online (2021) 43(3):395–403. doi: 10.1016/j.rbmo.2021.06.008 34332901

[23] Ferreux L Bourdon M Sallem A Santulli P Barraud-Lange V Le Foll N. Live Birth Rate Following Frozen-Thawed Blastocyst Transfer Is Higher With Blastocysts Expanded on Day 5 Than on Day 6. Hum Reprod (Oxf Engl) (2018) 33(3):390–8. doi: 10.1093/humrep/dey004 29394365

[24] Gardner D Lane M Stevens J Schlenker T Schoolcraft W. Blastocyst Score Affects Implantation and Pregnancy Outcome: Towards a Single Blastocyst Transfer. Fertil Steril (2000) 73(6):1155–8. doi: 10.1016/s0015-0282(00)00518-5 10856474

[25] Goto S Kadowaki T Tanaka S Hashimoto H Kokeguchi S Shiotani M. Prediction of Pregnancy Rate by Blastocyst Morphological Score and Age, Based on 1,488 Single Frozen-Thawed Blastocyst Transfer Cycles. Fertil Steril (2011) 95(3):948–52. doi: 10.1016/j.fertnstert.2010.06.067 20674914

[26] Richter K Harris D Daneshmand S Shapiro B. Quantitative Grading of a Human Blastocyst: Optimal Inner Cell Mass Size and Shape. Fertil Steril (2001) 76(6):1157–67. doi: 10.1016/s0015-0282(01)02870-9 11730744

[27] Ahlström A Westin C Wikland M Hardarson T. Prediction of Live Birth in Frozen-Thawed Single Blastocyst Transfer Cycles by Pre-Freeze and Post-Thaw Morphology. Hum Reprod (Oxf Engl) (2013) 28(5):1199–209. doi: 10.1093/humrep/det054 23477908

[28] Honnma H Baba T Sasaki M Hashiba Y Ohno H Fukunaga T. Trophectoderm Morphology Significantly Affects the Rates of Ongoing Pregnancy and Miscarriage in Frozen-Thawed Single-Blastocyst Transfer Cycle *In Vitro* Fertilization. Fertil Steril (2012) 98(2):361–7. doi: 10.1016/j.fertnstert.2012.05.014 22682029

[29] Ahlström A Westin C Reismer E Wikland M Hardarson T. Trophectoderm Morphology: An Important Parameter for Predicting Live Birth After Single Blastocyst Transfer. Hum Reprod (Oxf Engl) (2011) 26(12):3289–96. doi: 10.1093/humrep/der325 21972253

[30] Hill M Richter K Heitmann R Graham J Tucker M DeCherney A. Trophectoderm Grade Predicts Outcomes of Single-Blastocyst Transfers. Fertil Steril (2013) 99(5):1283–9.e1. doi: 10.1093/humrep/der325 23312233

[31] Tsampalas M Gridelet V Berndt S Foidart J Geenen V Perrier d'Hauterive S. Human Chorionic Gonadotropin: A Hormone With Immunological and Angiogenic Properties. J Reprod Immun (2010) 85(1):93–8. doi: 10.1016/j.jri.2009.11.008 20227765

