# Supplementary material for: Effect of Blastocyst Morphology and Developmental Rate on Euploidy and Live Birth Rates in Preimplantation Genetic Testing for Aneuploidy Cycles With Single-Embryo Transfer
Source: Front Endocrinol (Lausanne). 2022 Apr 13;13:858042. doi: 10.3389/fendo.2022.858042 (PMC9044033; doi:10.3389/fendo.2022.858042)
Supplement: Supplementary file 1 [file Image_1.pdf]

Supplementary Figure. Comparison of euploidy rates by blastocyst morphology and developmental rates in all age groups.

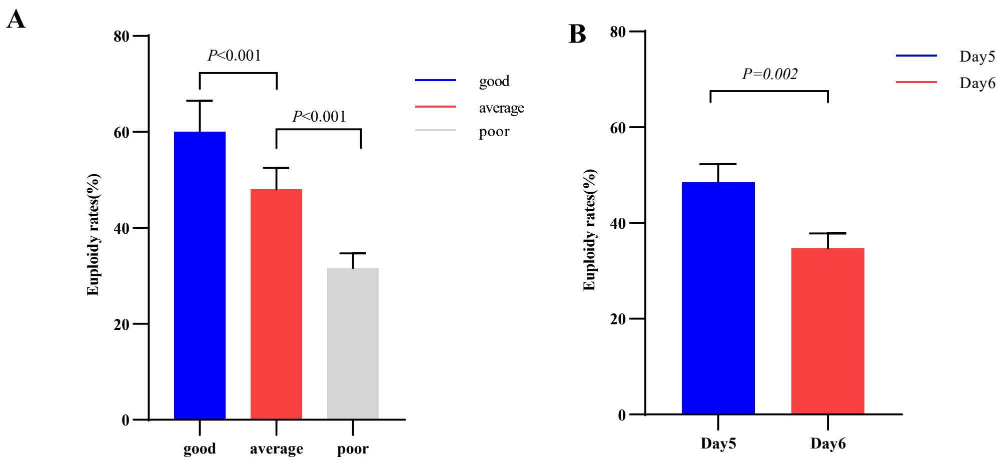

Note: Figure A, odds ratio was adjusted for blastocyst developmental rate, maternal age, maternal BMI, duration of infertility, type of infertility, infertility diagnosis, number of prior pregnancies, indication for PGT-A and basal FSH; Figure B, odds ratio was adjusted for blastocyst morphology, maternal age, maternal BMI, duration of infertility, type of infertility, infertility diagnosis, number of prior pregnancies, indication for PGT-A and basal FSH. *P* values are adjusted *p* values.
