# Supplementary material for: Effect of Blastocyst Morphology and Developmental Rate on Euploidy and Live Birth Rates in Preimplantation Genetic Testing for Aneuploidy Cycles With Single-Embryo Transfer
Source: Front Endocrinol (Lausanne). 2022 Apr 13;13:858042. doi: 10.3389/fendo.2022.858042 (PMC9044033; doi:10.3389/fendo.2022.858042)
Supplement: Supplementary file 2 [file Table_1.docx]

Supplementary Table. Generalized estimating equations analysis of the association between blastocyst morphology, developmental rate and euploidy.

| Variable | Value | Euploidy rate(%) | OR (95% CI) | *P* value |
| --- | --- | --- | --- | --- |
| Blastocyst |  |  |  |  |
|  | Good | 60.00 (135/225) | 2.900 (2.092–4.019) | <0.001 |
|  | Average | 47.98 (226/471) | 1.828 (1.439–2.323) | <0.001 |
|  | Poor | 31.64 (286/904) |  |  |
| Developmental rate |  |  |  |  |
|  | Day 5 | 48.49 (322/664) | 1.431 (1.139–1.798) | 0.002 |
|  | Day 6 | 34.72 (325/936) |  |  |

OR, odds ratio; CI, conﬁdence interval; The values for euploidy rates are adjusted for blastocyst morphology, developmental rate, maternal age, maternal BMI, duration of infertility, type of infertility, infertility diagnosis, number of prior pregnancies, indication for PGT-A and basal FSH.
